# Single-Point Incremental Forming of Titanium and Titanium Alloy Sheets

**DOI:** 10.3390/ma14216372

**Published:** 2021-10-25

**Authors:** Valentin Oleksik, Tomasz Trzepieciński, Marcin Szpunar, Łukasz Chodoła, Daniel Ficek, Ireneusz Szczęsny

**Affiliations:** 1 Faculty of Engineering, Lucian Blaga University of Sibiu, 550024 Sibiu, Romania; 2 Department of Manufacturing and Production Engineering, Faculty of Mechanical Engineering and Aerionautics, Rzeszow University of Technology, al. Powst. Warszawy 8, 35-959 Rzeszów, Poland; 3 Doctoral School of Engineering and Technical Sciences, Rzeszow University of Technology, al. Powst. Warszawy 12, 35-959 Rzeszów, Poland; d547@stud.prz.edu.pl; 4 Department of Integrated Design and Tribology Systems, Faculty of Mechanics and Technology, Rzeszow University of Technology, ul. Kwiatkowskiego 4, 37-450 Stalowa Wola, Poland; l.chodola@prz.edu.pl; 5 Department of Aerospace Engineering, Faculty of Mechanical Engineering and Aeronautics, Rzeszow University of Technology, al. Powst. Warszawy 8, 35-959 Rzeszów, Poland; ficekd@prz.edu.pl (D.F.); rm@prz.edu.pl (I.S.)

**Keywords:** friction, incremental sheet forming, lubrication, microstructure, sheet metal forming, single-point incremental forming

## Abstract

Incremental sheet forming of titanium and its alloys has a significant role in modern manufacturing techniques because it allows for the production of high-quality products with complex shapes at low production costs. Stamping processes are a major contributor to plastic working techniques in industries such as automotive, aerospace and medicine. This article reviews the development of the single-point incremental forming (SPIF) technique in titanium and its alloys. Problems of a tribological and microstructural nature that make it difficult to obtain components with the desired geometric and shape accuracy are discussed. Great emphasis is placed on current trends in SPIF of difficult-to-form α-, α + β- and β-type titanium alloys. Potential uses of SPIF for forming products in various industries are also indicated, with a particular focus on medical applications. The conclusions of the review provide a structured guideline for scientists and practitioners working on incremental forming of titanium and titanium alloy sheets. One of the ways to increase the formability and minimize the springback of titanium alloys is to treat them at elevated temperatures. The main approaches developed for introducing temperature into a workpiece are friction heating, electrical heating and laser heating. The selection of an appropriate lubricant is a key aspect of the forming process of titanium and its alloys, which exhibit unfavorable tribological properties such as high adhesion and a tendency to adhesive wear. A review of the literature showed that there are insufficient investigations into the synergistic effect of rotational speed and tool rotation direction on the surface roughness of workpieces.

## 1. Introduction

Titanium is the ninth most abundant element on Earth [1]. At the turn of the 20th and 21st centuries, titanium and its alloys were widely used in various industries such as the automotive, energy, chemical and food industries [2]. Components and entire structures made of titanium and its alloys can be found in particular in aviation and aerospace, where high structural strength and low weight are required [3].

Titanium alloys have a high yield stress of over 1550 MPa for Ti-(α + β) and β-type Ti-based alloys coupled with a relatively low density of 4600 kg/m^3^ [4]. The mechanical strength of titanium and its alloys ranges from R_m_ = 290 MPa for pure titanium Grade 1 to about 1750 MPa for heat treated β-type alloys [4]. The use of titanium alloys allows the strength of the structure to be increased while reducing the weight because titanium alloys have a density that is about 1.7 times lower than high-strength steels [5]. Titanium alloys have the highest specific strength up to a temperature of 600 K when compared to the most commonly used materials, such as steel and aluminum alloys. Titanium and its alloys are covered with a natural layer of titanium oxide TiO_2_, which makes these materials resistant to the effects of weather, sea water and many chemicals [5,6,7]. Titanium has a very low thermal conductivity coefficient, which is 22.08 W/(mK) [8], 13 times lower than that of aluminum.

According to ASTM B265, pure titanium is produced in 7 grades (Grades 1 to 4, 7, 11 and 12) that differing in their degree of contamination. The mechanical properties of titanium depend on its purity. Along with the increasing content of admixtures (Fe, O, N, C, Si and H), the formability decreases and at the same time the strength properties and hardness increase [9]. The mechanical properties of pure titanium can be changed by plastic working [9]. The effects of strain hardening can be eliminated by annealing recrystallisation. The yield stress of pure titanium varies from about 170 MPa (Grade 1) to 480 MPa (Grade 4), and the tensile strength from 240 MPa (Grade 1) to 550 MPa (Grade 4) [10].

**Figure 1:**
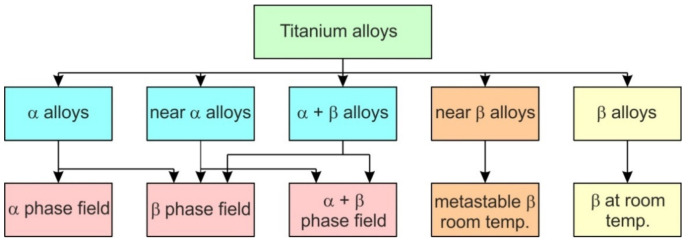
Classification of titanium-based alloys.

Titanium alloys crystallize in two crystalline structures and can be classified into an α-type (hexagonal-closed packed (HCP) crystalline structure stable up to the transformation temperature, near α-type, α + β, a β-type (body-centred cubic (BCC) crystalline structure stable from β-transus to melting point) and near β-type Ti-based alloys [11]. The transformation temperature can be changed by the addition of alloying elements [12]. The α-phase is stabilized by elements such as carbon, nitrogen and aluminum, while chromium, manganese, niobium, molybdenum and vanadium stabilize the β phase. The β-type Ti-based alloys show good plasticity but lower strength than α alloys [13,14]. The low modulus of elasticity of this group of materials ranges between 105 and 120 GPa. Depending on the ratio of the α- and β-type phases, Ti (α + β) alloys are subgrouped into near-α and near-β alloys (Figure 1) [15]. Due to the high content of elements stabilizing the β phase [16], and the slowing down of the aging process, β-type alloys have a low tendency toward work hardening.

The α–β alloy Ti-6Al-4V is the most popular of all titanium alloys, representing more than 50% of the titanium market [17]. However, wide usage of the Ti-6Al-4V sheet is limited by its poor formability at room-temperature [18]. The Ti-6Al-4V alloy can be solution heat-treated, quenched and aged to medium-/high-strength levels and have good formability due to the presence of the β-phase, which has a much higher diffusivity and more slip systems than α and near-α alloys [17]. For these reasons, TI-6Al-4V alloy is also more easily machined than other Ti-based alloys, and that is one of the reasons it is a widely used alloy. The Ti-6Al-4V alloy is designed for a good balance of characteristics, including strength, ductility, fracture toughness, high-temperature strength, creep characteristics, weldability, workability and thermal processability (higher strength is easily obtained by heat treatment) [19]. Based on Ti-6Al-4V, a significant number of titanium alloys have been developed by combining Ti-(4−6)Al with contents between 4% and 5% of β stabilizer elements, which include biomedical Ti-6Al-7Nb, Ti-5Al-2.5Fe, Ti-5Al-2Sn-2Zr-4Mo-4Cr, Ti-6Al-2Sn-4Zr-6Mo, Ti-6Al-6V-2Sn and Ti-7Al-4Mo alloys. New types of titanium, such as gamma alloys (TiAl phases, 46Ti–48Al alloy with Ti_3_Al), have been developed [15,20].

Conventional manufacture of metallic products relies on forging, casting and rolling of bulk feedstock materials, followed by subsequent machining to final shapes and dimensions [21]. These traditional manufacturing processes always inevitably result in a large amount of material waste, high manufacturing cost and long leading time [22]. Metal forming involves changing the shape of the material by permanent plastic deformation. Moreover, due to the work hardening phenomenon, the mechanical strength of the material formed increases. The advantages of the sheet metal forming (SMF) processes include no wastage of raw material, better mechanical properties of the product and faster production rate. SMF is used for large batches, which amortize tooling costs, producing large quantities of components during a short time interval. However, the possibility of using conventional stamping processes for small batches or personalized prototypes is very expensive [22]. A variant of the conventional SMF process, incremental sheet-forming (ISF) technology, presents an innovative possibility to decrease the cost of the problem in small volume production. It introduces the use of metallic sheet for small-batch production in an economic way without the need for expensive or dedicated tools [23]. Casting is a manufacturing process in which a liquid material is usually poured into a mold, which contains a hollow cavity of the desired shape, and is then allowed to solidify [24]. The casting products easily have various defects and deficiencies, such as inclusions, pores, shrinkages and cracks inside the casting components. The casting dimensional accuracy is low compared with that of machining components [24]. Machining of the titanium alloys is limited due to of the many machining difficulties occurring as a result of its hardness; chemical reactions that take place between the tool and the machine workpiece; and the high amount of heat generated in the cutting zone [25].

Plastic working of titanium alloy sheets has a significant role in modern techniques for the production of high-quality products with complex shapes [26,27]. Sheet metal forming constitutes a large element in the plastic processing of metals, which is the technique used for the creation of a wide range of components for the automotive, aviation and household appliances industries, as well as for the needs of biomedical engineering [28]. Due to the relatively limited production volume of a series of products made of titanium and its alloys, often for small series, incremental sheet-forming (ISF) methods, i.e., single-point incremental forming (SPIF) and two-point incremental forming (TPIF), are well-suited forming techniques. ISF has generally short forming times and low costs for forming sheet metal components because this process does not require special and costly die tooling.

The main advantages of SPIF and TPIF include [29,30,31]:

- The possibility of forming elements on a conventional CNC machine;
- Quick and easy consideration of design changes in the components being shaped;
- Significantly lower forming force values compared to conventional stamping;
- A higher value of the deformation limit of the sheet compared to conventional stamping.

The main disadvantage of SPIF is longer forming time compared to traditional methods of sheet metal forming; therefore, this method is economically justified for the production of components in piece and small-lot production [10,29,32]. SPIF and TPIF of titanium alloys have specific properties, the knowledge of which is necessary for the proper management of the process. Recent development trends in sheet metal forming and SPIF of lightweight metals have been provided by Trzepieciński et al. [33,34].

This article provides an overview of the development of incremental forming techniques for titanium and its alloys. In the following chapters, problems linked to the crystal lattice of titanium alloys and its tribological phenomena are considered. SPIF methods used in cold and elevated temperatures and SPIF for biomedical implants are discussed. One of the sections is devoted to the optimization of the forming strategy and the improvement of friction conditions when forming titanium and titanium alloy sheets. The scope of the review covers all applications of ISF methods for product forming, in particular in aircraft, automotive and medical applications.

## 2. Methods of Review

The Ei Compendex, IngentaConnect, PubMed, Scopus, ScienceDirect and Web of Science database search strategy used in this review was consistent with the principles of a systematic review according to the Preferred Reporting Items for Systematic Reviews and Meta-Analyses (PRISMA) [35] guidelines. The search strategy was limited to articles written in English. To ensure the high quality and timeliness of the review, great emphasis was placed on searching for both review and research papers, review papers, books, chapters and conference proceedings, over the last ten years. Overall, the following numbers of papers were of interest to the authors: 160 research papers in scientific journals, 20 review papers in scientific journals, 10 books, 20 chapters and 30 papers in conference proceedings. On the basis of the titles and abstracts of the bibliographical sources found, an initial selection of articles was carried out, based on the heat-assisted incremental sheet forming method, and on the main purpose of the article (springback analysis, analysis of surface roughness, lubrication conditions, etc.). Articles, the content of which, despite a promising summary, differed from the assumed subject of this review article, were excluded from further analysis. Obviously, duplicate articles found in above-mentioned databases have been removed. In addition, articles by the same authors were reviewed for similar or duplicate content. This approach was often used when publishing extended versions of conference materials in scientific journals. 

## 3. Incremental Sheet Forming Methods

**Figure 2:**
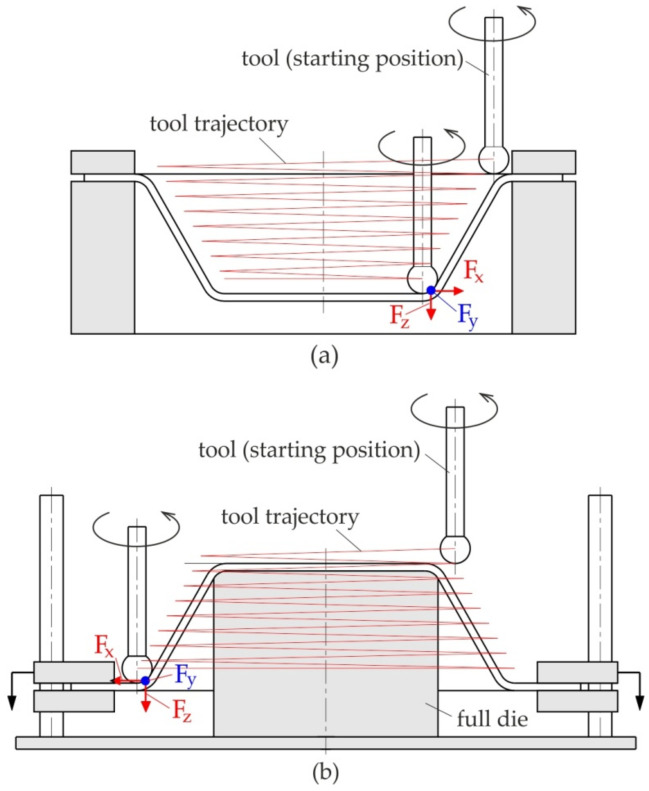
(**a**) Negative and (**b**) positive ISF.

SPIF and TPIF involve the gradual forming of sheet metal using rotating contour tools. Due to the penetration of the tool producing a stretch process in accordance with the assumed trajectory (forming strategy) of the tool movements, the deformed material takes on a final shape through the accumulation of localized plastic deformation of the sheet, in which a hemispherical-head tool controlled by a computer program is used to form the metal [36,37,38]. Incremental forming is classified into two main varieties:

- Negative (Figure 2a), in which there is an additional element of the tool in the form of a template or a pin supporting the highest area of the detail being formed; forming takes place by appropriate mapping of the template geometry, by “arranging” the sheet on it, with a tool performing programmed movements,
- Positive (Figure 2b), in which the component is formed by the movement of the tool in accordance with the tool trajectory without the supporting template.

The definition of positive and negative is given by the concavity of the part that is shaped, which can be concave up or concave down.

**Figure 3:**
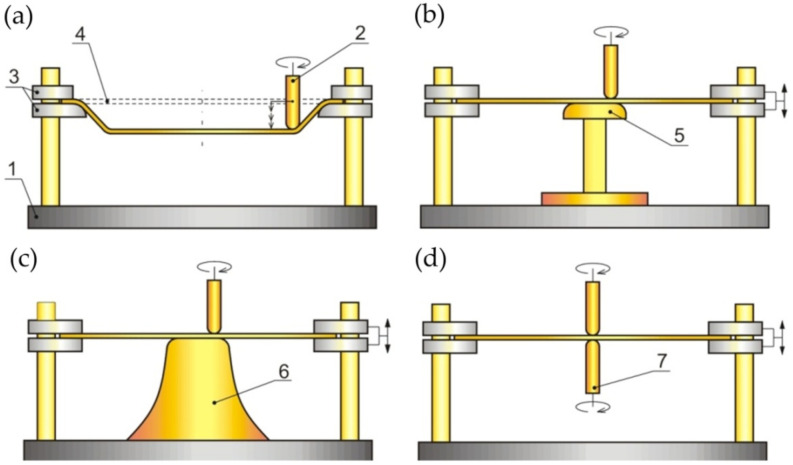
ISF Processes: (**a**) SPIF; (**b**) TPIF with a partial die; (**c**) TPIF a specific die; and (**d**) IFWCT: 1—clamping device, 2—forming tool, 3—plate holder, 4—workpiece (initial position), 5—partial die, 6—specific die and 7—auxiliary forming tool.

**Figure 4:**
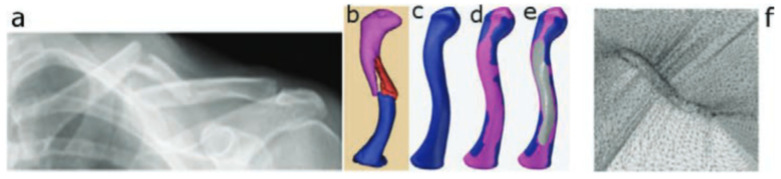
(**a**) Computer tomography scan of a clavicle, (**b**) 3D reconstruction, (**c**) reconstructed clavicle, (**d**) clavicle with predicted muscle attachment sites (blue area), (**e**) clavicle with designed implant and (**f**) implant embedded in extension structure (reproduced with permission from [74]; copyright © 2017 Published by Elsevier Ltd.).

Due to the kinematics of the tools, incremental forming has several varieties that differ in the equipment used. The first type is SPIF; the principle of carrying it out is shown in Figure 3a. The tool is guided by the machine control system along a pre-programmed trajectory, causing incremental deformation of the sheet [39,40]. A variation of SPIF (Figure 4a) is TPIF, which can be performed with the use of a partial die (Figure 3b) or a specific matrix (Figure 3c). The last method increases the geometric accuracy of the formed elements. In two-point forming methods, there is an additional movement of the assembly fixing the edges of the formed sheet, which translates into greater accuracy of the components. In the incremental forming with counter tool (IFWCT) method, an additional mandrel is placed opposite the forming tool and shifted by the thickness of the sheet moves along an appropriately corrected trajectory in relation to the main tool (Figure 3d).

Recently, the micro-ISF (μ-ISF) technique developed by Saotome and Okamoto [41] has received increasing attention due to its high operability and flexibility, and it is regarded as a very promising technique for micro-structure manufacturing [42,43]. Μ-ISF was developed by the use of the scanning electron microscope (SEM) to achieve in situ observation of the forming process. Although the μ-ISF process increases flexibility and reduces installation costs, it presents many problems related to significant shape defects and large decreases in thickness [44].

## 4. Incremental Sheet Forming towards Biomedical Applications

Titanium is considered to be the most biocompatible of the metals due to its resistance to corrosion in body fluids and bio-inertness [10,45,46]. The strength of titanium is due to a protective oxide film that forms naturally in the presence of oxygen found in body fluids [47]. The oxidized coating adheres strongly to the tissue and is insoluble and chemically impermeable, which prevents it from reacting with the surrounding environment [48]. The biological neutrality of titanium is used in surgery in the form of implants, i.e., spinal implants, dental implants and knee or hip endoprostheses [49,50]. Recent developments in β-type Ti-based alloys for biomedical applications have been provided by Chen et al. [51].

Biocompatibility is defined as the ability of a material to behave properly in contact with biological tissue. Titanium is a metal that additionally exhibits so-called osseointegration, i.e., the ability to create a direct structural connection between its surface and living tissue [52,53,54]. As a result, no scar tissue or cartilage is formed between bone and the implant, and the connection is very strong. Diffusive oxynitride layers improve the hemocompatibility of titanium and its alloys, including its antithrombogenocity [55,56].

More than 1000 Mg of titanium is implanted in patients worldwide each year [57]. The implant materials must be non-toxic and non-carcinogenic and have similar mechanical properties to the original bones [58,59]. The appropriate geometric profile of the implant and the quality of the surface after forming are guarantees of biocompatibility [60,61,62]. The implant should be adapted to the authorized requirements of each patient, so the processing of implants is basically a unit production process [63]. Under these conditions, the use of ISF methods seems to be ideal. Since the sheet metal is formed incrementally, the formability and potential of material can be fully explored [64,65]. A number of efforts have been made to manufacture implants and supports for different parts of the human body using ISF, such as the skull [66,67,68,69], dental prostheses, knee [70], face [71] and ankle support [72].

Through the years, researchers have investigated and proved several aspects of the process feasibility of using SPIF for manufacturing biomedical implants. Han et al. [73] successfully produced a prosthesis for a skull on a titanium mesh plate, which can increase the contact area and adjust the elasticity modulus of the material. Duflou et al. [66] found that during ISF of a cranio-facial implant, the limit forming angle of pure titanium is 47°. In order to increase this limit, a multi-stage forming strategy was proposed, and the results showed that the limit forming angle was increased to 61°. Duflou et al. made an effort to use ISF to manufacture cranial reconstruction from titanium Grade 2 [66]. This paper discusses the challenges associated with the manufacture of cranio-facial implants with extreme forming angles. Vanhove et al. [74] fabricated titanium Grade 2 thin-shell clavicle implants through SPIF. The fracture-fixation and bone-aligning tasks of these implants call for specific accuracy distribution, while the distinct geometry and post-forming heat treatment influence the production accuracy. The authors have shown the potential to replace bulk generic clavicle implants (Figure 4) by successfully compensating for the forming toolpath in order for it to be brought into a satisfactory accuracy range. 

Sbayti et al. [18] evaluated the feasibility of producing a customized acetabular component of a hip prosthesis with an acceptable geometric accuracy using this process and based on the use of Ti-6Al-4V titanium alloy. It was found that the geometric accuracy was significantly improved at T ≥ 400 °C. Castelan et al. [57] manufactured custom-made cranial implants from a sheet of Grade 2 titanium using 3D printing, CAD/CAM technology and SPIF. Araújo et al. [75] evaluated the SPIF process for the fabrication of customized maxillofacial implants from titanium and demonstrated the role of this technology in manufacturing customized medical parts. Ambrogio et al. [76] manufactured the Ti-6Al-4V and Ti6Al4V-ELI prostheses by SPIF. The authors designed a complete procedure that guides and integrates the jobs of surgeons and engineers during the cranioplasty process. Cytotoxicity tests have shown that SPIF does not affect the biocompatibility of the prostheses. 

**Figure 5:**
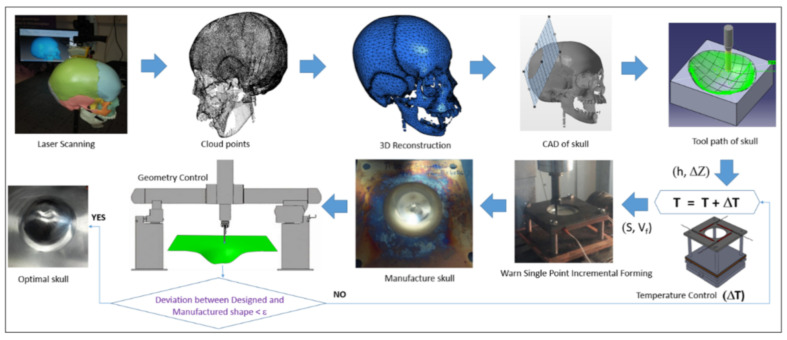
A reverse engineering application to human skull manufacture (reprinted with permission from [79]; copyright © 2018, Springer-Verlag London Ltd., part of Springer Nature).

**Figure 6:**
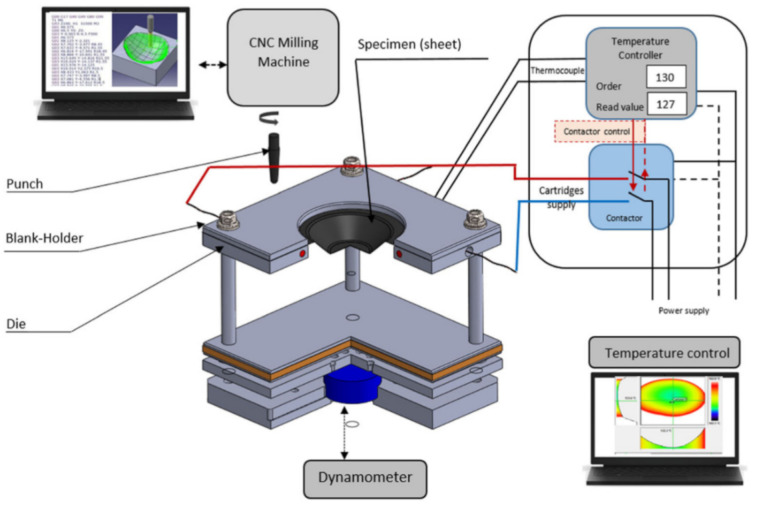
Scheme for warm SPIF (reprinted with permission from [79]; copyright © 2018, Springer-Verlag London Ltd., part of Springer Nature).

Many solutions have been proposed by Honarpisheh et al. [77] and Mohammadi et al. [78] in order to overcome the formability limitation of titanium alloy in warm SPIF. Results have shown that increasing the working temperature is very effective in decreasing the elastic springback and forming force and improving formability. Saidi et al. [79] developed a reverse engineering approach (Figure 5) associated with a warm SPIF process (Figure 6) in order to produce a Ti-6Al-4V prosthesis of a human skull. The major advantages of this heating system compared to others proposed in the literature are its low cost, great simplicity of installation and limited increase of equipment technology.

Castelan et al. [57] physically constructed a CAD model of a skull with 3D Printing, and SPIF was used to manufacture the implant from titanium Grade 2. It was found that dimensional variability can be reduced with changes in the manufacturing process (i.e., forming and cutting) and the heating ramp. Before cutting the final shape of the implant, heat treatment was performed to avoid deformations caused by residual stresses generated during the SPIF.

**Figure 7:**
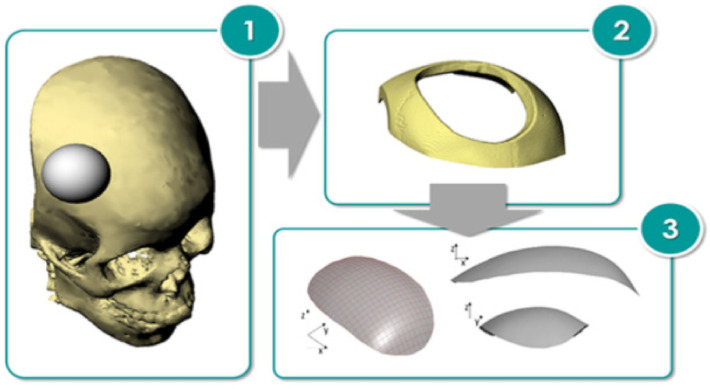
Design process of the prosthesis geometry (reproduced with permission from [80]; copyright © 2020 The Authors; this is an open-access article distributed under the terms of the Creative Commons CC BY license, which permits unrestricted use, distribution and reproduction in any medium, provided the original work is properly cited).

Palumbo et al. [80] fabricated prototypes of Ti-Gr5 and Ti-Gr23 cranial prostheses via the single-point incremental forming process. The geometry of the prosthesis investigated was obtained by starting from a model of a human skull in poly (methyl methacrylate) material (Figure 7). A defect was created by removing a portion of the skull, and finally the shape of the prosthesis was thus defined by reverse engineering. The comparison between finite element-based simulation numerical data and experimental data revealed that the performance in terms of impact response of the prostheses strongly depends on its thickness distribution, due to the strain hardening phenomena.

Araújo et al. [71] SPIFed facial implants with titanium Grade 2. The main operating parameters influencing the production of facial implants with SPIF were identified. Investigations demonstrated how circle grid analysis combined with the fracture forming limit diagram (FLD) can be utilized to successfully re-engineer the fabrication of a facial implant. The results show how the overall level of in-plane strains and drawing angles can be controlled in order to obtain a sound facial implant produced by SPIF.

Oleksik et al. [81] presented a study based on experimental research on the surface quality of the medical implants used for the partial resurfacing of the femoral condylar surface of the knee obtained by SPIF. The research examined the punch diameter, the friction conditions and the initial roughness of the punch between the punch and the blank.

## 5. Thermally-Assisted Incremental Sheet Forming

The hot incremental forming process provides the opportunity to improve the formability of Ti alloys [33]. It is almost impossible to plastic-form Ti alloys at room temperature because of the hexagonal close packed structure of the alpha phase and major plastic deformation by twinning [82]. When increasing the forming temperature, proper lubrication is a key factor affecting the surface roughness obtained, as well as the oxidation process [83]. An elevated process temperature can be provided by different heat sources [84]. In the last two decades, researchers have focused on elevating the temperature of Ti alloy sheets by: direct current (DC) and alternating current (AC), cartridge heaters, induction, laser source, and friction between the tool and the workpiece. Heat sources can be arranged to cover just the precise location [85], the whole sheet or a combination of both. Local heating is energy-efficient, and the sheet oxidation zone is reduced. However, deformation is only enhanced at the tool-workpiece contact zone. Grade 5 seems to be the most favorable for selection for heat-assisted ISF because of its wide application in the aerospace, automotive and medical industries; light weight; strength; and biocompatibility. The method that has been most intensely studied in the last two decades seems to be electrically-assisted ISF due to the low cost of the apparatus needed to upgrade the conventional forming test stand.

In general, heating systems in thermal-assisted ISF processes can be classified as [86]:

- Radiation—a laser light follows the tool trajectory (the workpiece is heated at the actual location of forming) [87,88,89],
- Convection—hot air blowers heat the entire metal sheet [90],
- Conduction—heater bands are mounted on the external surface of the die to heat the entire workpiece during ISF [91],
- Electricity—an electric current is used to heat the workpiece at the actual location of forming [77,92,93],
- Friction—material is heated due to frictional forces between the rotating tool and the fixed sheet [94,95,96].

### 5.1. Electric Heating

**Figure 8:**
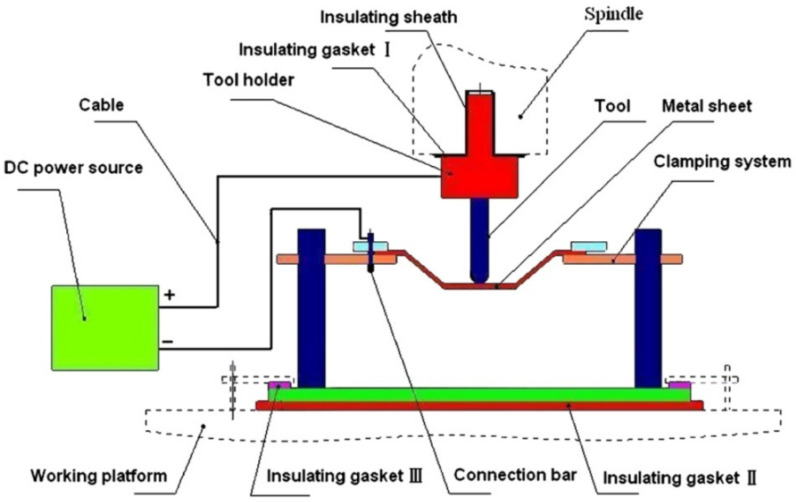
The principle of electric incremental forming (reproduced with permission from [92]; copyright © 2008 Elsevier Ltd. All rights reserved).

Fan et al. [92] were some of the first researchers to apply electric DC current to heat TiAl2Mn1.5 sheet. By connecting the tool to the cathode and the sheet to the anode of the power source, according to Joule’s law Q = I^2^·R·t (Q is the heat generated, I is the current, R is the resistance of the conductor and t is the time), electricity heats at the location of the contact point in the deformation zone (Figure 8). It was found that applying a 400 A current with a feed rate of 400 and 800 mm/min, using hemispherical tools with diameters of 8, 10 and 12 mm and step sizes of 0.1 and 0.2 mm, allows one to form a 50° wall angle cone, a distorted tetragonal pyramid and a tetragonal pyramid shape.

**Figure 9:**
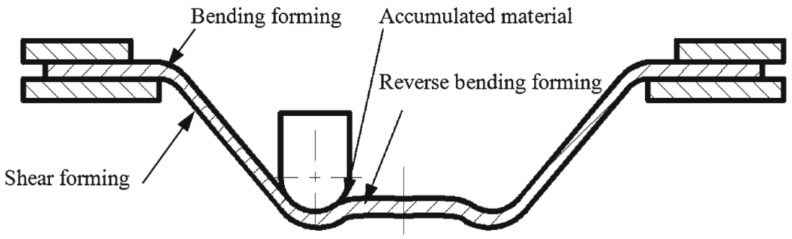
Forming regions in a SPIFed component (reprinted with permission from [93]; copyright © 2014, Springer-Verlag London).

In further work, Fan et al. [83] used this method to form Ti Grade 5 with a 400 A current and feed rate of 15 mm/s, achieving a sheet temperature range of 300–500 °C and resulting in a maximum forming angle of 72°. Improved formability due to electric pulses could be affected by a reduction of dislocation density and a boost to its mobility [97]. In addition, a weakened base texture and weakened anisotropy leads to improved formability [98]. Asgar et al. [99] investigated the DC pulse assisted by TPIF process, where the cathode is connected to the upper punch tool and the anode to the bottom, while the tool moves independently. A 0.6-mm-thick titanium alloy sheet can be successfully formed into a pyramidal shape with angles of 30° and 45° using 140 A current. The springback effect was significantly reduced by this method, while it required about twice the forming force compared to the no current variant. As Fan and Gao [100] proved, with a higher feed rate, with a constant step size and wall angle, a higher DC current should be applied to maintain an elevated temperature. Heat level feedback and a proper heat source controller are required to maintain a constant forming temperature. Metallographic structure analysis shows that hot electric-assisted ISF is a process where plastic hardening and annealing occur. To achieve a uniform structure, heat treatment is necessary after forming. Elevated temperatures decrease the tensile strength of Ti Grade 5 due to oxidation, so a protective gas should be applied. Fan and Gao [93] investigated the electric hot ISF of Ti Grade 5 experimentally and compared this to numerical simulation using MSC.Marc software. Simulation revealed three zones of deformation: bending, which takes place at the beginning; shear forming in the middle; and reverse bending at the end. Furthermore, to enhance accuracy and reduce bending during the formation of the first layer, adequate support is required. Fan and Gao [93] also discuss the different regions (bending deformation at the beginning, shear forming in the middle and reverse bending at the end) in assuring the geometric accuracy of the SPIFed components (Figure 9).

**Figure 10:**
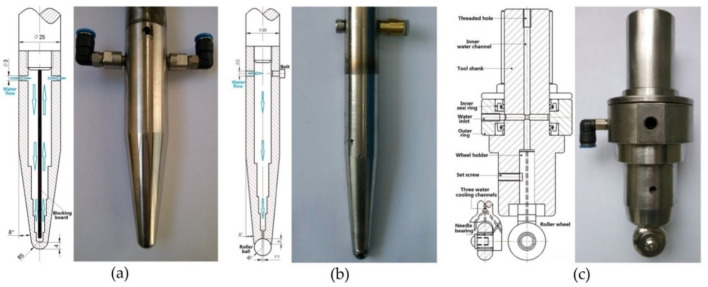
The tools that have been developed for use in E-ISF: (**a**) conventional rigid tool with inner water-cooling channel, (**b**) rolling-ball tool with inner water-cooling channel and (**c**) rolling-wheel tool with inner water-cooling channel (reprinted with permission from [101]; copyright © 2015, Springer-Verlag London).

To understand the process a little better, the Johnson–Cook Finite Element Method (FEM) model finds good agreement with experiment during electric hot incremental forming (EHIF) of Ti Grade 5 [77]. Elevated temperature in the contact zone has effects in accelerating the tool wear and on the final surface quality of the part. To reduce the impact of temperature during the EHIF of Grade 5, Liu et al. [101] developed an advanced cooling system integrated with forming tools (Figure 10), equipped additionally with a freely rolling tip. This solution allows the tool temperature to be reduced from over 500 °C to 100 °C and the tip wear from 1 mm to less than 0.1 mm when compared with conventional tools. By this approach, the sheet surface parameter has also been enhanced from Ra = 2.939 µm, Rz = 17.226 µm to Ra = 0.805 µm, Rz = 4.64 µm.

**Figure 11:**
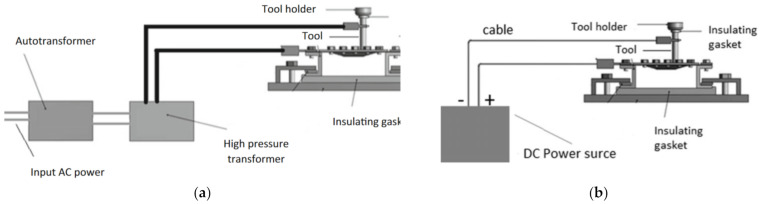
Schematic diagram of the E-ISF process with (**a**) AC and (**b**) DC power (reprinted with permission from [102]; copyright © 2016, Springer-Verlag London).

Najafabady and Ghaei [102] developed a novel approach to the process by using AC (Figure 11a) current instead of DC (Figure 11b) for safety reasons (less sparks and easier to break the circuit). An autotransformer and high-pressure transformer have been employed to adjust the voltage from the 220V electric network to 1–1.5 V and deliver a current of 100–200 A. In their work on Ti Grade 5, 0.62 and 1.05 mm thick constant wall angle conical frustums (CWACF), varying wall angle conical frustums (VWACF) and two types of pyramidal frustum have been experimentally investigated within the 150–200 A range of currents, with a 600 mm/min feed rate, 100 and 600 rpm tool rotational speeds, 0.1 and 0.3 mm step sizes and a target temperature of 600 °C.

**Figure 12:**
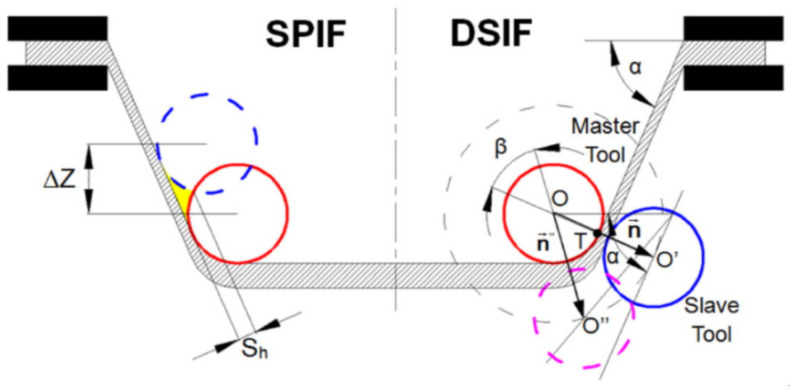
Comparison between tool paths in SPIF and DSIF (reproduced with permission from [103]; copyright © 2015 Elsevier Ltd. All rights reserved).

Increasing the step size from 0.1 to 0.3 mm produced a rise in the inside surface Ra parameter (from 6.8 µm to 11.2 µm). Furthermore, a thicker sheet blank produced an improved outer surface Ra parameter (from 3.2 µm to 0.8 µm). Proper lubrication is a key factor in enhancing surface quality at elevated temperatures. By spraying MoS_2_ powder, the Ra parameter has been reduced from above 13 µm under dry conditions to less than 5 µm. A micro hardness test revealed a rise from 291 HV at the flange to 350 HV at the vertex caused by the work-hardening effect. Maximum shape deviation was observed in the workpiece flange with a tendency to decrease towards the vertex—this effect can be significantly reduced in the Electrically-assisted Double Sided Incremental Forming (E-DSIF) process [103]. Figure 12 shows the toolpath strategies utilized.

**Figure 13:**
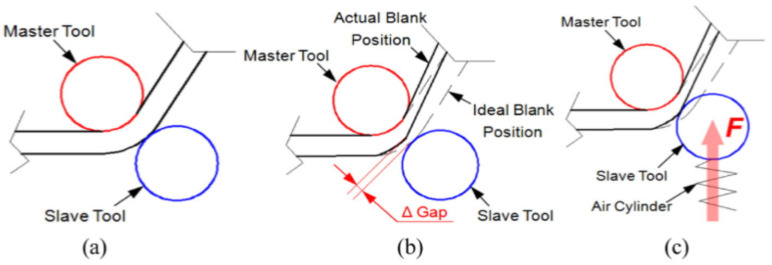
The E-DSIF process: (**a**) ideal position of forming tools, (**b**) loss of contact of slave tool and (**c**) supported slave tool approach to ensure a stable contact (reproduced with permission from [103]; copyright © 2015 Elsevier Ltd. All rights reserved).

The general concept of DSIF is shown in Figure 13a, in which the sheet is deformed by the motions of tools on both sides. Conventional DSIF approaches usually rigidly control the displacement of both master and slave tools and may result in loss of contact between the tool and the sheet due to sheet thinning, as shown in Figure 13b. Therefore, Xu et al. [103] proposed a device to ensure stable contact between the tool and the sheet, where the slave tool is supported by an air cylinder, which acts as a spring (Figure 13c).

Ortiz et al. [104] conducted an experiment where 10kW static heaters were located below the Ti Grade 5 1.6 mm thick sheet. In VWACF, in which the forming angle increases constantly, the angle limit was determined at a furnace temperature of 800 and 900 °C, feed rate of 1000 mm/min, and step size of 0.5 and 1.0 mm. The forming depth limits and angles obtained were 25.4 mm (45°) for room temperature, 44 mm (55°) at 800 °C and 72 mm (70°) at 900 °C. Obviously, VWACF is not sufficient to define the forming limit; subsequently, a constant 70° wall angle conical frustum of 35.3 mm depth was successfully CWACF-formed until breakage. In further work, Ortiz et al. [105] presented the intelligent process model (IPM) to correct the tool path, which improved the model accuracy in response to springback by up to 49%. Khazaali and Fereshteh-Saniee [106] conducted a groove test with the Taguchi method. Three static heaters have been placed below, forming Grade 5 sheet at an elevated initial temperature of up to 190 °C. A tool diameter of 16 mm and a step size of 0.7 mm at 190 °C seem to be the most favorable parameters from the experimental range. In further experiments [107], the authors extended their research on VWACF by conducting 27 experiments (3 levels) using the Taguchi method with three temperature levels (100, 200 and 300 °C), tool diameters (8, 12 and 16 mm), and step sizes (0.4, 0.7 and 1.0 mm). Optimum measurements of the maximum drawing depth—300 °C, Ø8 mm tool and 1mm step size—were determined. Saidi et al. [79] investigated the manufacture of a cranial part from Grade 5 sheet by incremental forming using cartridge heaters as a heat source. In their work, an elevated temperature from 30 °C to 130 °C produced at least a doubling in the reduction in maximum vertical force and of average deviation from the desirable shape. Mohanraj and Elangovan [108] heated up the punching tool to 300 °C in their work using DC resistance. The optimum forming parameters of 300 °C, 0.1 mm step size and 50° wall angle were found for Grade 2. Furthermore, the theoretical thermal model proposed by the authors agrees with the experiment conducted.

### 5.2. Induction Heating

**Figure 14:**
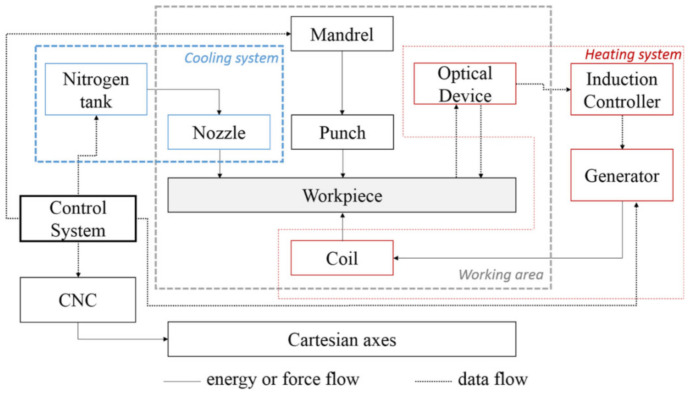
Functional scheme of the experimental equipment (reprinted with permission from [86]; copyright © 2016, Springer-Verlag London).

Ambrogio et al. [86] proposed induction heating 1mm thick nonmagnetic Grade 5 to 600 and 700 °C with cryogenic cooling. The induction coil was located below the sheet and follows the tool path. For cryogenic cooling, liquid nitrogen supplied at 6 bar was sprayed axially onto the forming tool to cause quenching. The cryogenic medium was sprayed while the punch retraced from the last coil. A schematic illustration of the equipment used is presented in Figure 14. During the experiment, cryogenic versus standalone ambient cooling was compared. The induction heating system has an advantage over DC electric heating in terms of sheet surface roughness. The authors obtained an Ra from less than 2.5 µm to over Ra 4 µm in the 1mm step size forming test. Due to cryogenic cooling, the Grade 5 grain growth process was avoided and the micro hardness remained unchanged.

Gagliardi et al. [91] noted that in order to avoid the coil overheating, a low- to medium-frequency generator with high power is more suitable for non-magnetic sheets, such as Grade 5. Low-medium frequency would be important with regard to process feasibility to ensure that it is not detrimental to the duration of coil working life, while at the same time it increases the heating capacity of the system.

### 5.3. Laser Heating

Göttmann et al. [87] applied a laser beam to provide localized heat to Grade 2 and Grade 5 during asymmetric incremental sheet forming, in order to compare differences between the heat assisted and the room temperature setup. With experimental parameters of a laser beam with 1700 W power, step size of 0.35 mm, forming velocity of 4000 mm/min and two variants of tool diameter (20 and 30 mm), Grade 5 appears to show significant improvement in forming, although Grade 2 has not improved formability causing thermal failure. To achieve constant temperature in the contact zone, one can place an inside forming tool with a thermocouple, and a PD device could be adopted to control the source power of the laser or the DC electric current. Forming forces are also stabilized by this closed loop steering of temperature [88]. In the work conducted by Mosecker et al. [89], an ISF experiment was extended using a constant laser power source and by adding air cooling through a nozzle behind the tool path. The microstructure changes of Grade 5 and micro hardness differences have also been taken into account. The mainly observed deformation mechanism was the dynamic recovery, but at a temperature of 850 °C, without cooling, dynamic recrystallisation appeared, as well as the deepest deformation obtained. However, at this range of temperatures, Grade 5 micro hardness increases due to the grain size and reduction in the sheet thickness. This effect can be suppressed by applying dynamic recrystallisation cooling.

### 5.4. Friction Heating

**Figure 15:**
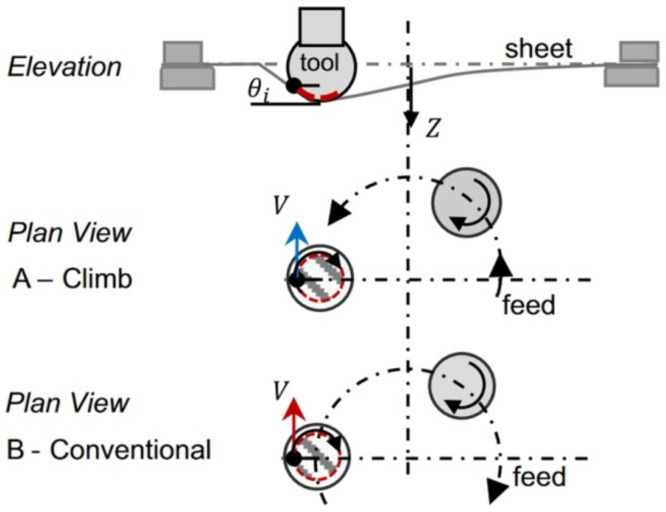
The concept of conventional and climb SPIF (reproduced with permission from [96]; copyright © 2018, The Author(s); this is an open-access article distributed under the terms of the Creative Commons CC BY license, which permits unrestricted use, distribution and reproduction in any medium, provided the original work is properly cited).

Friction stir incremental forming is a type of localized heat initiation method in which heat is generated by friction between the tool and the workpiece due to the high-speed rotary movement of a punch. In work by Ambrogio et al. [94], titanium Grade 2 and Ti-based Grade 5 alloy were examined by applying high-speed single-point incremental forming. A lathe CNC machine was used as a test stand rather than the generally selected CNC milling machines. This was due to the fact that asymmetrical parts cannot be manufactured using the adopted speed (2500 m/min) because of the feed rate limits of the currently produced CNC milling machines. However, they allow for a quick manufacturing process—a frustum cone of 30° for Grade 2 and 25° for Grade 5 (Ø180 mm upper and Ø42 mm lower diameter) has been successfully formed in less than 1 min. Grün et al. [95] investigated design of experiment (DoE) and One Factor At a Time (OFAT) tests with various tool rotational speeds, feed rates and step sizes during forming VWACF from 1 mm thick Grade 5 using a Ø11 mm ball-ended tool. A major factor affecting formability and the value of the forming forces is tool rpm, while tool feed rate and step size are less significant. Elevated rotation speeds decrease forming forces by increasing contact temperature, although they rapidly increase tool wear and remove material from the sheet surface. The relationship to feed rate rotation direction, climb or ‘conventional’ (see Figure 15) is also a key parameter, as Uheida et al. [96] investigated in VWACF of Grade 2.

Conventional type forming has higher forming temperatures and lower vertical and horizontal forces, leading to better accuracy and a higher forming angle in comparison to climbing. However, the conventional forming direction produced poor surface quality due to increased friction causing the material to overheat.

### 5.5. Combined Electrical and Friction Heating

Palumbo and Brandizzi [109] investigated the heating of Grade 5 sheet by tool rotation in the range of 800–1600 rpm and additionally replaced the electric static heating with 3000 W electric bands. Electric heating warms up the whole blank, while the tool rotation only heats the location of the tool-blank contact zone. By increasing the tool rotational speed, the sheet surface parameter Ra worsens, but necking is stabilized. Khazaali and Fereshteh-Saniee [110] investigated the groove test as a benchmark of process parameters, in order to determine FLDs, reduce the blank size and reduce the experiment time. In their work, heating elements were placed below the Grade 5 blank from 0.5 mm thick sheet. The sheet temperature needed to be compensated for by a pyrometer and in fact was different from that obtained with the heaters. With constant process parameters of 400 rpm for the tool; 400 mm/min feed rate; and variable step size (0.1, 0.4 and 0.7 mm), temperature (100, 200 and 300 °C) and tool diameter (8, 12 and 16 mm), 27 trials were performed. In conclusion, elevated temperature, larger tool diameter and step size are critical to obtaining improved forming. It was also noted that higher temperature and tool diameter reduces the springback effect and increases shape accuracy—especially between the sheet clamping area and the formed shape (angular difference from 21.2°, 100 °C, tool Ø8 mm to 12.7° for 300 °C, and Ø16 mm tool).

## 6. Accuracy, Springback Reduction and Toolpath Optimization

Studies on increasing the accuracy of parts produced by SPIF from pure titanium or titanium alloys can be classified in studies that are focused on determining the optimal values of process parameters such as punch diameter, vertical step, friction coefficient, punch speed and tool rotation direction. The studies on springback reduction are focused on finding new technological improvements such as electrically-assisted SPIF, hot-forming SPIF, or DSIF. Toolpath optimization techniques are generally related to improving accuracy and reducing springback and involve finding different algorithms in order to correct the initial toolpath.

Palumbo et al. [111] studied the behavior of the titanium alloy Ti-6Al-4V, an alloy with a strong anisotropy, during single-point incremental forming. The research was based on the numerical simulation of this procedure using the Abaqus/Explicit analysis program. To conduct the numerical research, the mechanical properties and those measured during the uniaxial tensile test were determined for specimens taken at 0°, 45° and 90°, by means of the Aramis 3D optical measurement system. This allowed for the anisotropy of the titanium alloy, as well as its influence on SPIF, to be highlighted. The results were focused on determining the plastic strains and the thinning and shape errors. In the first step, to analyze the influence of the main input parameters on SPIF, a truncated pyramid was chosen as the geometric model for the part manufactured by SPIF. The yield criterion chosen for the titanium alloy was Hill (1948). A face-centered central composite design (CCD) was built in order to perform the numerical simulations. The two input parameters of the SPIF process considered were the ratio between the punch diameter and the vertical step (D/pz) and the wall angle (θ). Regarding the geometric precision, the authors concluded that the parts with the smallest shape errors are obtained when the ratio between the punch diameter and the vertical step (D/pz) has the highest values. Based on the data obtained from the first stage results, the research continued for a car door shell, with two values for the D/pz ratio: 3 and 16. A higher value of D/pz was observed to lead not only to a better geometric accuracy but also to a reduction of thinning on the parts obtained.

Ambrogio et al. [94] analyzed the influence of the punch speed on the behavior of two materials, Grade 2 and Ti-6Al-4V, during SPIF. Speeds between 5 and 500 mm/min were used for Grade 2 and between 6 and 600 mm/min for the titanium alloy. The vertical pitch used varied for both types of materials from 0.1 to 1 mm. The authors showed that it is possible to increase the speed of the punch and implicitly reduce the manufacturing time (the SPIF process being known to be time consuming) without producing a significant degradation of the surface quality. With the increase of punch speed on the Ti-6Al-4 V alloy, a 2% increase in micro-hardness is observed. However, the authors of the study concluded that using a small vertical step leads to long manufacturing times but also to an increase in temperature due to repeated friction between the punch and the blank.

**Figure 16:**
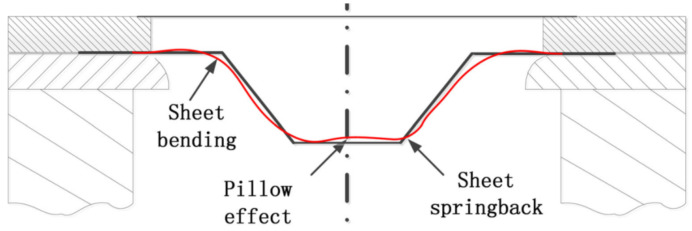
Geometric errors during the SPIF process (reproduced with permission from [113]; copyright © 2007 Elsevier B.V. All rights reserved).

Sbayti et al. [112] propose an algorithm for optimizing the SPIF process factors (punch diameter, vertical step and friction coefficient) to obtain a denture plate with the purpose of increasing its geometric accuracy. Their research is based on simulation using the finite element method and the material used to make the denture plate is Ti Grade 1. The algorithm presented by the study authors starts with the CAD configuration of the geometric model of the denture plate and continues with toolpath generation, numerical simulation using the finite element method, error measurement and the optimization of the process factors, to reduce the geometric errors of the part. The response surface methodology was used as an optimization method, with two optimization techniques having been applied: the single objective optimization technique and the multiple-objective optimization technique. The Box–Behnken strategy was used to develop the design of experiments. In the optimization process for the process factors, all three major groups of geometric errors that occur in parts manufactured by SPIF were taken into account, namely: the springback error, the pillow effect error and the bending error (Figure 16). 

The mathematical expressions for the three types of errors were determined in the form of second-degree polynomials using a variance analysis (ANOVA) [112]. Main effect plots for the punch diameter, vertical step and friction coefficient were also graphically presented. A genetic algorithm (GA) was used for the single objective optimization technique, which allows for error minimization for one of each type of the above-mentioned errors (springback error, pillow effect error and bending error). For the multiple-objective optimization technique, which allows for the simultaneous minimization of the three geometric errors being considered, a combination of GA, global optimum determination by linking and interchanging kindred evaluators algorithm (GODLIKE), and grasshopper optimization algorithm (GOA) was used [113]. The numerical simulations were subsequently validated by experimental tests obtaining the optimal values for the three process factors.

**Figure 17:**
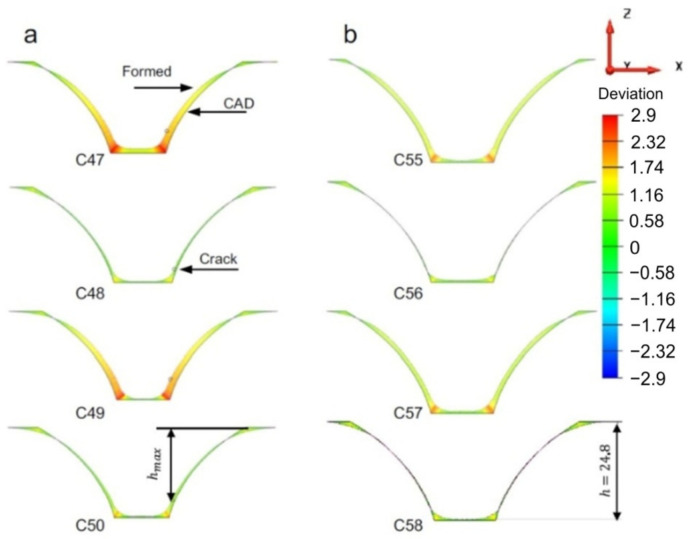
Deviation of the cross-sectional profiles with (**a**) climbing rotation and (**b**) conventional rotation at different heights in the part (reproduced with permission from [96]; copyright © 2018, The Author(s); this is an open-access article distributed under the terms of the Creative Commons CC BY license, which permits unrestricted use, distribution and reproduction in any medium, provided the original work is properly cited).

The effect of the tool rotation direction on the formability, surface quality and geometric accuracy of parts made of pure titanium sheets was analyzed by Uheida et al. [96]. The study, based on experimental research, was performed on axisymmetric parts with a variable angle (from 30° to 75°), using a spiral trajectory and a 10 mm diameter punch. Both a “conventional rotation” tool direction, in which the spindle rotation and the feed direction are coincident (both in a clockwise direction), and a “climbing rotation”, in which the spindle rotation remains in a clockwise direction while the feed direction is changed to a counter-clockwise direction, were used. To evaluate the accuracy of the parts obtained, 2D contours of the inner surface were measured at different heights with the help of a coordinate measuring machine, which were subsequently used for the 3D reconstruction of the geometric model. The cross-sectional profiles obtained for the two variants are presented in Figure 17. Once the model was reconstructed, it was compared with the CAD model in order to determine the springback errors. It has been found that both types of tool rotation direction lead to parts with a lower height than desired and that a correction of the trajectory in the sense of an “overform” is necessary to reduce this error. However, the shape deviation that appears in proximity to the cone-opening flanges, also called bending error at SPIF, is instead influenced by the tool rotation direction with the smallest errors found to occur in parts manufactured using the “conventional” tool rotation direction.

A combined study—numerical, using the finite element method, and experimental, to improve the accuracy of titanium parts manufactured by electric hot incremental forming—was presented by Fan and Gao [93]. One of the most-used titanium alloys, Ti-6Al-4V, an alloy with very low elongation and high strength that is processed by forming only at high temperatures, was used in this study. Due to the pronounced elasticity of this alloy, the springback errors are higher than with SPIF manufacturing of other types of materials [114]. The first stage consisted of simulation during incremental forming at elevated temperature for a pyramid-type frustum part with a wall angle of 50°, using the MSC-Marc program for the numerical simulation. The electric hot incremental forming process is particular in that the temperature is not uniform on the whole part but produces local heating in the area immediately close to the punch. This feature was introduced in the simulation as the heat transfer rate and was determined following the analysis of the equivalent thermal strain, equivalent plastic strain, and stresses. The authors of the study concluded that, unlike SPIF manufacturing of other metals or alloys in which the predominant deformation was the shear deformation, electric hot incremental forming presents three types of deformation: bending deformation in the first stage, shear deformation in the middle stage and reverse bending in the last stage. Another conclusion drawn by the authors was that the equivalent plastic strain only appears starting with the fourth vertical step due to the low temperature and then increases rapidly. This is the reason the geometric accuracy of the part is influenced by the first trajectories and the reverse bending is influenced by the plastic strain. In order to reduce the reverse bending, the authors propose the use of two point incremental forming together with electric hot incremental forming.

**Figure 18:**
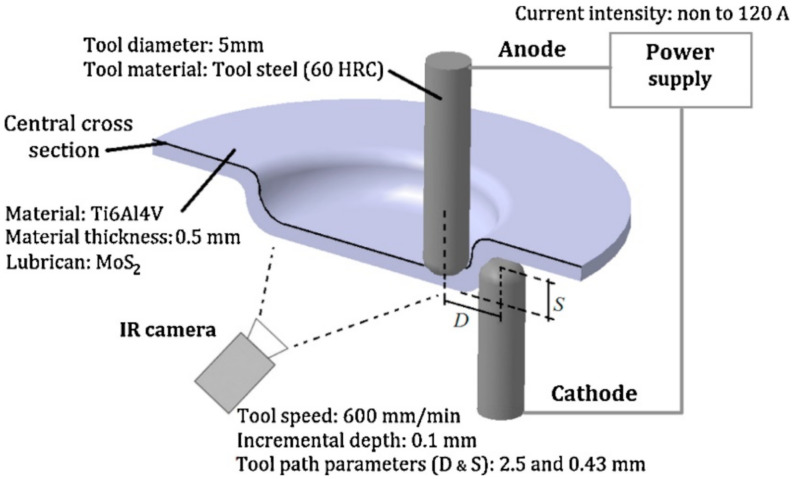
The experimental layout used to study the differences between electrically-assisted cumulative double-sided incremental forming (E-ADSIF) and electrically-assisted mixed double-sided incremental forming (E-MDSIF) (reproduced with permission from [115]; copyright © 2016 College International pour la Recherche en Productique).

Valoppi et al. [115] conducted a comparative study between electrically-assisted cumulative double-sided incremental forming (E-ADSIF) and electrically-assisted mixed double-sided incremental forming (E-MDSIF), in forming Ti-6Al-4V titanium alloy. The experimental layout used is shown in Figure 18. 

A double curvature part was the geometry chosen for the study, and the vertical step used was of 0.1 mm. The main purpose of the paper [115] was to study the influence of electricity on the formability of titanium alloy, but the geometric accuracy and roughness of the parts obtained was also analyzed. For E-ADSIF, the authors used different intensities of electric current, ranging from 40 A to 120 A, and for the E-MDSIF they used the optimal intensity obtained in the case of E-ADSIF. In order to estimate the geometric accuracy, the parts were measured with a non-contact laser instrument and were compared with the ideal geometry. Thus, for E-ADSIF the best results in terms of accuracy were obtained at a current intensity of 50 A. However, at the same current intensity of 50 A, but instead using E-MDSIF, the accuracy is significantly improved.

Another paper that analyzes the behavior of the Ti-6Al-4V alloy during hot single-point incremental forming is presented by Saidi et al. [116]. The novelty factor of this study is that the authors are replacing the expensive laser heating systems that perform local heating on the forming area with a cheaper alternative based on heat cartridges. This variant allows for the heating of the entire surface of the blanksheet, leading to increased formability. In parallel with experimental research, numerical simulations were also performed using the finite element method, with the authors recording small differences (of up to 2%) between the experimental and numerical results. In order to highlight the geometric accuracy of the cone-shaped parts when a frustum wall angle of 55° is obtained, the part profiles were measured and compared with the ideal profiles. The areas with the largest geometric deviations were the bending zone (the connection area between the flange and the wall element); the wall area, where the springback effect occurs; and the bottom of the part, where the pillow effect occurs. The maximum deviation value appeared in the bending zone, and the maximum springback value appeared in the area where the maximum thickness reduction was also recorded.

Behera and Ou [117] analyzed the influence of stress-relieving heat treatment on the geometric accuracy and surface topography of titanium parts manufactured by incremental forming. In the case of pure titanium and titanium alloys, residual stresses appeared and remained during forming operations, unfavorably affecting the subsequent mechanical behavior of the part. Because of this, it is essential to remove these residual stresses by applying the stress-relieving heat treatment. The aforementioned study used titanium Grade 1 with a 0.5 mm thickness. Following SPIF manufacturing and the application of heat treatment (before or after the forming process), the parts obtained were subjected to X-ray diffraction in order to highlight the effect of the stress-relieving heat treatment, as well as being subjected to an analysis using a 3D interferometer in order to study the surface topography. The authors observed the appearance on the surface of the part of an “orange peel effect”, which is characteristic of titanium, but also a worsening of the roughness by 0.5 μm on the free surface of the part (the surface that does not come into contact with the punch). The geometric accuracy of the parts was analyzed both before and after the trimming process, which is necessary in order to extract the useful area of the part. The conclusions of the study show that applying the stress-relieving heat treatment before SPIF leads to both an improvement of the surface roughness, as well as to an increase in the geometric accuracy of the parts obtained. Additionally, the value of the residual stresses is lower in the case of the parts that were subjected to stress-relieving heat treatment than in the case of those that have not undergone this treatment at all.

Han et al. [73] developed a digital manufacturing technology for a skull prosthesis based on the fact that creating medical implants and a prosthesis requires an individualized manufacturing model, with each model having its own geometry. Thus, starting with the images from Computed Tomography (CT) and using software for the reconstruction of CAD models based on Non-Uniform Rational B-Splines (NURBS), the authors obtained the geometry of the skull prosthesis. Based on this geometry, the authors went on to generate the tool path using industrial software, followed by the optimization of the punch trajectory in order to reduce errors and springback.

Another paper that studies the technological variants of manufacturing of cranial implants by SPIF on the basis of an analytic hierarchy process (AHP) is that of Racz et al. [118]. For the analytic hierarchy process, they chose four types of manufacturing technologies, including three related to incremental forming, namely, conventional SPIF, hot SPIF and DSIF. Seven criteria were chosen for evaluation using the AHP: formability, microstructure, degree of control, roughness, energy consumption, accuracy and production time. They used three types of tool path for the SPIF manufacturing of implants: two circular, one of which had vertical feed points on the same generatrix, the second of which had other vertical feed points indexed at different angles, and a third spiral trajectory. A profilometer was used to analyze the geometric accuracy of the implants obtained, and the main strains and thickness reduction were determined by means of an optical system. Following this analysis, the authors found the spiral trajectory to be the optimal option for the manufacturing of complex parts such as cranial implants.

**Figure 19:**
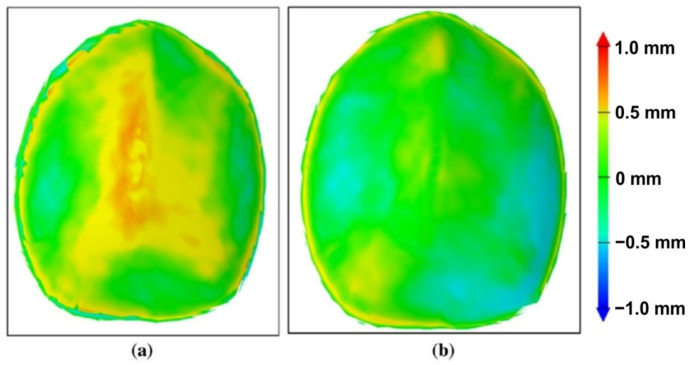
The accuracy plot of a cranial implant manufactured by SPIF with (**a**) an uncompensated toolpath and (**b**) a compensated toolpath (reproduced with permission from [119]; copyright © 2015, Springer-Verlag London; this is an open-access article distributed under the terms of the Creative Commons CC BY license, which permits unrestricted use, distribution and reproduction in any medium, provided the original work is properly cited).

The dimensional accuracy of implants made of medical grade titanium and manufactured by SPIF was analyzed by Behera et al. [119]. They also proposed a method to improve the geometric accuracy by compensating the tool path. The authors considered the division of the geometric model into features of great importance, especially in the case of complex shaped parts with double curvature, such as cranial implants. The algorithm they presented contains, in summary, the following steps: the uncompensated toolpath is created starting with the CAD model of the part; the part is manufactured with this tool path and is then measured by means of an optical measurement system; the measurement leads to obtaining a point cloud, which is subsequently transformed into a mesh, and this mesh is compared to the initial CAD model, thus obtaining the deviations on the entire surface of the part. The accuracy file, an STL file, is then obtained. A software product created by the authors of the study identifies the features in this file and then links this file to a set of files called the training set CAD model. The linked data set is then exported to a data file for generating an accuracy response surface using multivariate adaptive regression splines (MARS). The compensated tool path is then obtained. In the study cited, the authors applied the abovementioned algorithm for both an ellipsoid part and a cranial plate. The results obtained for the cranial plate are presented in Figure 19. Using this algorithm led to obtaining a mean deviation of ±0.5 mm, a significant improvement compared to that of the cranial plate manufactured with the uncompensated toolpath.

**Figure 20:**
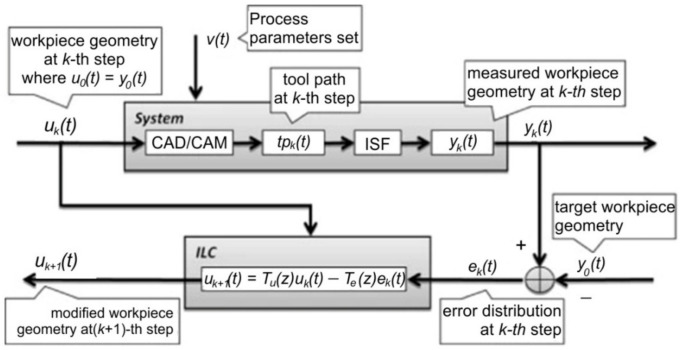
The algorithm used for increasing part accuracy: *u_k_(t)*—part geometry, *u_k+1_(t)*—part geometry (corrected), *v_k_(t)*—process parameters set, *tp_k_(t)*—toolpath, *y_k_(t)* —obtained geometry, *y_d_(t)*—target geometry, *e_k_(t)*—error map and *T_e_(z)*—correction weight (reprinted with permission from [120]; copyright © 2016, Springer-Verlag France).

A very interesting study, also based on the finite element method, was developed by Fiorentino et al. [120]. The study uses iterative learning control to increase the accuracy of Ti Grade 2 alloy parts manufactured by SPIF. Iterative learning control uses the following algorithm: the punch trajectory is determined, the part is manufactured, it is measured and the errors are determined. Once these errors are known, the punch trajectory is corrected in order to reduce the geometric error. This algorithm is then cyclically repeated until convergence, which is represented by obtaining a minimum imposed geometric error. The algorithm used by the iterative learning control is presented in Figure 20. The authors focused on parts with planar and curvilinear geometries with wall inclinations of (0°, 29° and 38°) and a depth of 30 mm. They also conducted a comparative study between three types of materials: aluminum Al 1050A, steel DC04 and titanium Grade 2 alloy. The iterative learning control algorithm was applied to the finite element method, and the validation of the results was done by experimental research on a CNC machine. Of all the three types of materials, the largest geometric errors (±1.1 mm) were obtained for titanium Grade 2 due to the springback phenomenon, which is most pronounced in the case of titanium alloys.

Improving the accuracy of industrial parts manufactured by SPIF is desirable, but improving the accuracy of medical implants is an extremely important goal. Thus, Duflou et al. [66] published some of the first concerns related to increasing the geometric accuracy of a frontal orbit cranial implant made of Grade 2. They additionally presented a comparative study between the conventional implant technologies and the single-point incremental forming process, also naming the advantages and disadvantages of the different types of technologies. The steps required to manufacture an implant by SPIF are: carrying out a CT scan of the injured skull, preparing a clay model of the skull, building a CAD file of the implant based on reverse engineering and producing the final 3D CAD model of the implant integrated into the blank sheet. The strategy used to make the implant is a multi-step tool path that allows for the formability of the material to be increased by redistributing the material. To evaluate the geometric accuracy of the orbit cranial implant, they scanned the entire implant following the SPIF processing, noting that there are some overformed areas, such as the backing plate, as well as underformed areas. For this reason, to compensate for the trajectory, an uncompensated trajectory was chosen for a model made of AA 1050 and was translated proportionally with the deviation obtained and multiplied by a scale factor equal to 0.7. By using this strategy, the geometric accuracy increased significantly, with the average deviation decreasing from 1.19 to 0.377 mm.

Li and Mo [121] studied springback in double curvature parts made of Grade 4 titanium alloy and manufactured by SPIF, based on a numerical simulation using the finite element method. As an example, the authors chose to use SPIF to manufacture a part of an aircraft skin. Using the geometry of the part, the authors inserted an addition that will later be removed by trimming in order to reduce springback. To estimate the springback, the authors presented the nodal displacement in three different sections of the part (two at the ends of the part and one in the middle), following the forming process before and after the appearance of springback. The conclusion of the study is that the plastic deformation value in the case of titanium parts is low, while the springback has particularly high values, especially in the middle area of the part and slightly lower towards the ends of the part. Additionally, the vertical feed values must be as low as possible in order to reduce the springback value and increase the accuracy.

**Figure 21:**
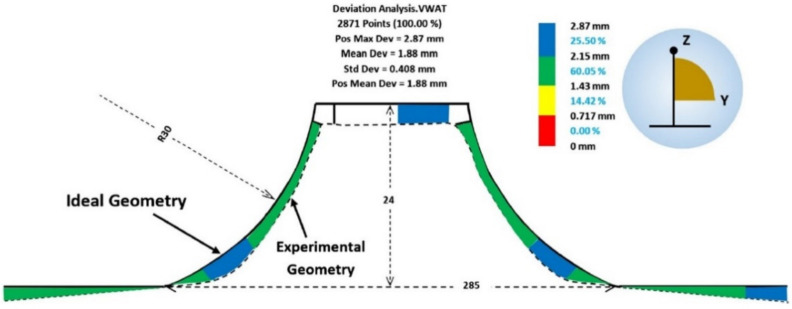
Deviation analysis for a truncated cone geometry (reprinted with permission from [102]; copyright © 2016, Springer-Verlag London).

Najafabady and Ghaei [102] conducted a study on the geometric accuracy, surface quality and hardness of titanium alloy parts produced by SPIF. There are other studies related to hot SPIF of titanium alloy, but the novelty brought by the authors is provided by the fact that the local heating due to the electrical contact is replaced by an electric heater. The main advantage is the use of alternating current electricity instead of direct current, with the alternating current allowing the use of cheaper and safer equipment for the operator. The authors studied the main areas where, due to the elasticity of the titanium alloy, the maximum deviations occur. A deviation analysis for a truncated cone geometry is presented in Figure 21. In this type of manufacturing, with the use of an electric heater, the maximum deviation occurs at the connection area between the flanges and the vertical walls, i.e., exactly in the starting area of the trajectory, due to the fact that in this area the blanksheet fails to reach the optimum temperature. After several vertical steps, this inconvenience disappears and the geometric accuracy increases.

**Figure 22:**
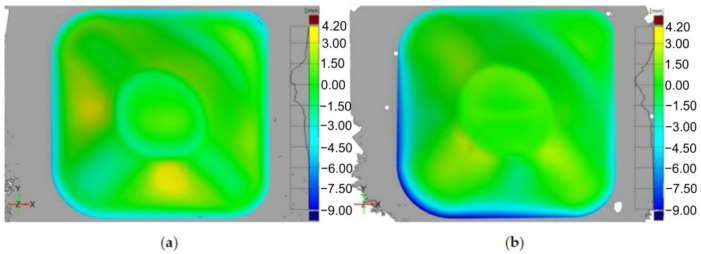
Accuracy analysis for (**a**) an uncompensated trajectory and (**b**) for a trajectory compensated with the intelligent modelling (reproduced with permission from [105]; copyright © 2015, The Authors; this is an open-access article distributed under the terms of the Creative Commons CC BY license, which permits unrestricted use, distribution and reproduction in any medium, provided the original work is properly cited).

Another paper refers to SPIF manufacturing accompanied by heating of the blanksheet of the Ti-6Al-4V titanium alloy [105]. Unlike other studies in which heating only occurs locally, near the forming area, the authors of this paper heated the whole blanksheet and kept the temperature constant throughout the entire punch movement. The main objective of the study was to improve the geometric accuracy by means of two approaches: using a trajectory that is subjected to a correction process in order to eliminate springback errors and reducing the deflection of the sheet along the perimeter part by adding an addendum to the shape of the part (in fact a connection between the tilted walls of the part and the undeformed, flat area). The authors studied the influence of part temperature and the trajectory on geometric accuracy. When considering the influence of temperature, it was found that, even if the shape deviation follows the same pattern, its value has a different influence on different areas of the part. Increasing the temperature leads to a large variation in wall angle, while decreasing the temperature leads to the reduction of deviations in the flat/low curvature areas. The trajectory correction was achieved by applying an intelligent process model that has the ability to predict the springback value and apply it to the initial CAD model, which thus modifies the new trajectory being obtained on this corrected geometric model. The use of this algorithm led to significant reduction of springback in the titanium part, as we can observe in Figure 22.

Grimm et al. [122] aimed to reduce springback during incremental forming of titanium alloys by electrically assisting this deformation process. They identified the residual stresses that remain inside the material after the forming process as the main cause of springback in the case of material-forming processes in general and of SPIF in particular. The authors also classified the factors influencing springback, namely, part geometry, part material and part thickness. Having taken all these into consideration, the authors centered their study on truncated pyramid shaped parts made of thick AMS-T-9046 titanium sheets with a thickness of 0.508 mm. Three test parts were initially manufactured, without the use of electricity, and these were then measured and compared with the ideal CAD models for the determination of springback. The same methodology was used for the manufacturing of parts in the presence of electricity, with several paths for applying electricity. The general conclusion was that the use of electrically assisted SPIF led to a significant reduction in springback in the case of AMS-T-9046 titanium sheets but also that the path the electricity takes in each part has an important influence on the reduction of springback.

## 7. Surface Quality

### 7.1. Forming Strategy

**Figure 23:**
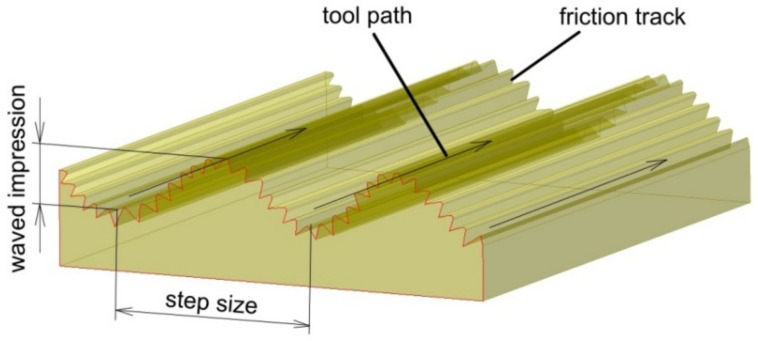
Surface of the drawpiece across the tool-path.

Large-scale waviness (Figure 23) created by the tool path is considered a weak point for ISF. The problem of poor surface quality of the SPIFed parts due to large scale waviness created by the tool path can be overcome in SPIF by optimization of the forming strategy. Waved impression roughness is the major surface quality problem of SPIF since the roughness value of the waved impression is larger than with the friction trace [123,124].

Recently, many studies have focused on applying different strategies to improve the geometric accuracy of finished parts made by SPIF, i.e., neural network strategies [125,126], iterative algorithms methodology [127,128] and the transfer functions approach [129].

Ortiz et al. [105] proposed two approaches to improving the geometric accuracy of typical aerospace Ti-6Al-4V parts produced by hot SPIF. In the first approach, it was proposed to skip the overforming deviations associated with the deflection of the sheet along the perimeter of the part based on a design improvement. In the second approach, an IPM was adopted that counteracts deviations associated with the elastic deformations of the component after springback. The IPM approach leads to an accuracy improvement of the tetragonal pyramid of up to 49%. Veeraajay [130] experimentally studied the effect of the feed rate, incremental step depth, spindle speed and tool feed on the surface roughness of Ti-6Al-4V truncated cones. Response surface methodology (RSM) was used to design the experiments, and ANOVA was performed to find the factor that significantly affects the surface finish of drawpieces. It was found that with an increasing feed rate, the surface roughness decreases from 1.05 mm to 0.9 mm. A better surface finish is achieved by decreasing the step depth.

**Figure 24:**
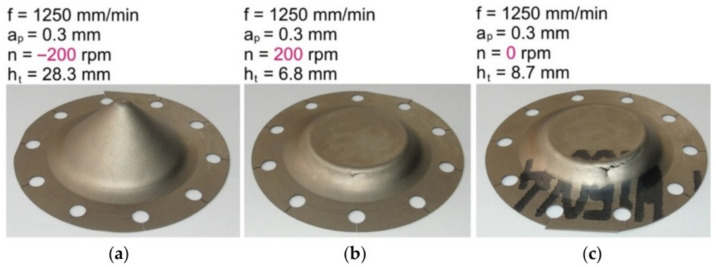
Grade 2 drawpieces formed at a constant step size of 0.3 mm, tool feed rate of 1250 mm/min and rotational speeds: (**a**) −200 rpm (clockwise direction), (**b**) 200 rpm (anticlockwise direction) and (**c**) freely rotatable tool).

Szpunar et al. [131] applied a central composite design to determine the mathematical model of the effect of tool feed rate, spindle speed and step size on the surface roughness of truncated cones of Grade 2 commercially pure titanium. It was found that samples formed with high values of spindle speed showed poor surface qualities. They also found that the direction of tool rotation in relation to the feed direction is one of the crucial parameters influencing the risk of cracking (Figure 24) [131]. Step down showed the strongest influence on surface roughness, which is concordant with the results of the Box–Behnken design analyzed by Yao et al. [127].

A decision-making method based upon an analytic hierarchy process was applied by Racz et al. [118] to optimize the SPIF parameters. The surface roughness of the SPIFed Ti-6Al-4V cranioplasty plates was directly influenced by the vertical step, a decrease in the vertical step leading to a decrease in the roughness value [118].

**Figure 25:**
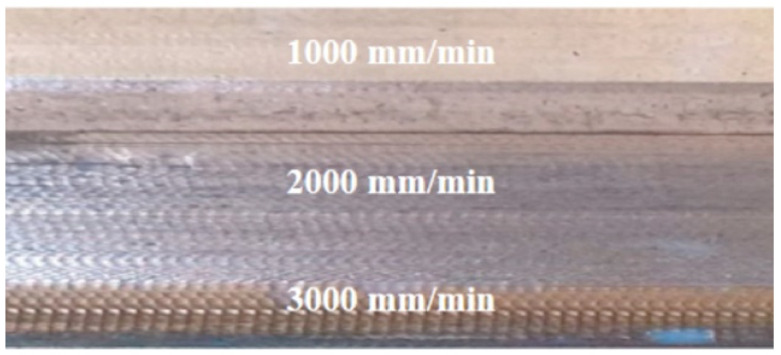
Images showing the effect of the feed rate on the surface quality of the sheet (reproduced with permission from [132]; copyright © 2019 The Author(s). Published by Elsevier B.V.).

The radius of the tool tip is one of the most important parameters that influences the surface finish of Grade 1 and Ti-6Al-4V sheets [132]. The higher the feed rate is, the poorer the surface quality of the workpiece is (Figure 25). Mohanraj and Elangovan [133] found that the negative incremental forming method with a sliding motion of the tool provides a better surface finish of Ti-6Al-4V. Ambrogio et al. [94] investigated the effect of feed rate with respect to the surface quality of formed Grade 2 and Ti-6Al-4V sheets in order to develop a high-speed SPIF machine. Preliminary analyses show that the impact of high-speed forming on the forming accuracy and surface roughness was not significant.

### 7.2. Friction Conditions

Titanium alloys have low wear resistance, high coefficient of friction (COF), a tendency to galling and relatively low hardness [134,135]. Therefore, the improvement of friction conditions is a key factor for surface finish [136,137]. The tribological properties of titanium and its alloys, in particular the tendency to seize during cold and hot forming operations, are intensified by high contact loads. Another significant problem occurring during the forming of titanium is the formation of sticking on the surface of tools (galling), which significantly affects the quality of manufactured products [138,139,140]. The unfavorable phenomena accompanying the SPIF and TPIF of titanium alloys can be limited by appropriate surface treatment of the tools and the use of appropriately selected technological lubricants [141,142]. Due to the fact that most of the lubricants used in sheet metal forming (SMF) processes belong to the group of synthetic oils, in recent years research has been conducted on replacing mineral oils with biodegradable vegetable oils [143,144], including those with the addition of nanopowders [145,146,147]. Trzepieciński et al. [144] tested the tribological properties of Ti-6Al-4V titanium alloy sheets in a strip drawing test using five kinds of vegetable oils: palm oil, rapeseed oil, olive oil, sunflower oil and soybean oil. It was found that increasing the load at a constant value of kinematic viscosity of lubricant reduces the value of the coefficient of friction (COF). The lowest value of COF was provided by oil with a low density and at the same time high kinematic viscosity.

Appropriate lubrication is essential to prevent the formation of an oxide layer on the inner surface of the part which promotes sheet fracture and tool wear [148,149]. Due to the high temperature of the sheet in hot SPIF, liquid lubricants cannot be used and therefore solid lubricants should be used instead [92,150].

A poor friction condition could lead to severe scratch, which can damage the implant surface. Moreover, heavy-duty contact conditions can lead to a temperature increase in the contact area. In particular, for titanium and titanium alloy, the adhesion phenomenon is more likely to happen under improper contact conditions [136]. Changing the contact conditions and improving the lubrication are currently the two main approaches adopted: modification of the tool and selection of lubricants with appropriate properties adapted to the processing conditions. Table 1 shows the most important lubricants used during SPIF or TPIF of titanium and Ti-based alloys. It was found that the molybdenum disulphide MoS_2_ grease is the most popular lubricant when forming this group of materials. 

**Figure 26:**
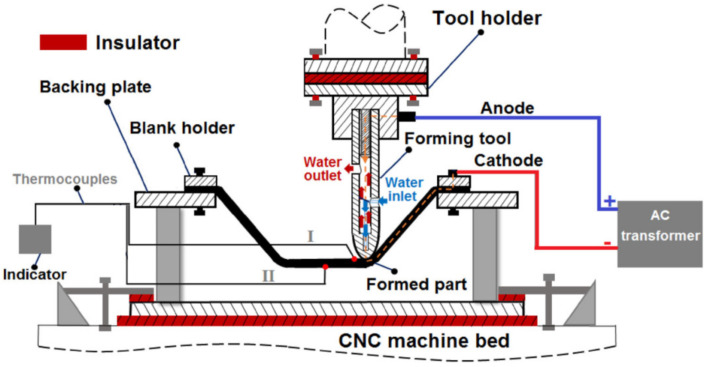
Schematic diagram of resistance SPIF (reprinted with permission from [150]; copyright © 2019, The Indian Institute of Metals—IIM).

Vahdani et al. [150] examined MoS_2_, graphite powder, and graphite-based and copper-based anti-seize lubricants. They experimentally investigated the effects of four different lubricants together with the influence of vertical pitch, feed rate and electric current on the surface finish of Ti-6Al-4V sheet in resistance SPIF using a forming tool with a cooling system that they had developed (Figure 26). It was found that the lubricant type has the most effect on the formability followed by the electric current. The best surface finish is obtained when using a graphite-based compound, owing to the more uniform distribution of the graphite particles within the grease.

Graphite is a good high-temperature lubricant, but when used in EHIF, electric sparks appeared between the tool and sheet metal, which may cause the creation of micro-pits on the surface of the sheet metal and tool [83].

MoS_2_ is often dispersed in metal matrices like copper and nickel to achieve enhanced strength of the lubricant for good wear resistance. Fan et al. [83] successfully formed Ti-6Al-4V alloy using EHIF at 500–600 °C with a small amount of oxidation. They confirmed that a self-lubricating Ni disulphide metal matrix composite is suitable for electric hot incremental forming at high temperature.

**Figure 27:**
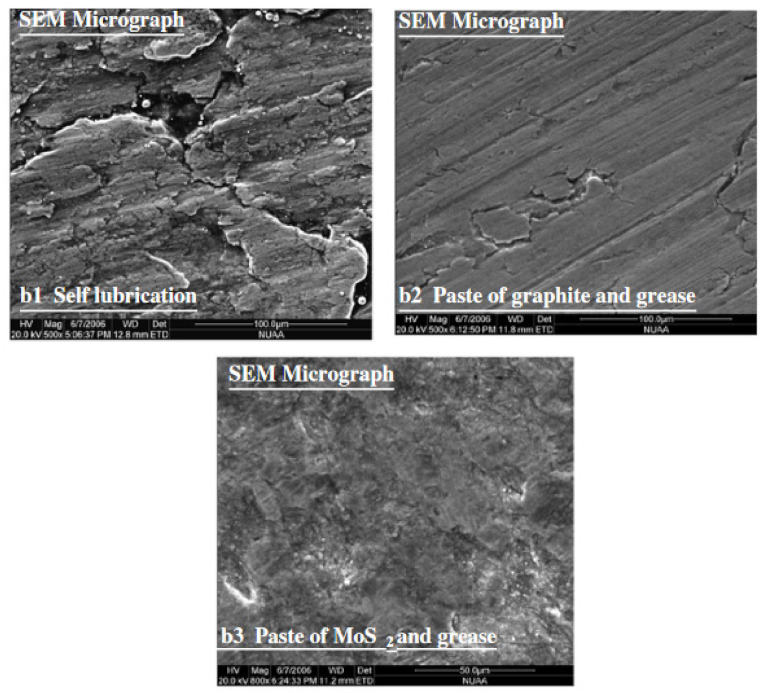
The effect of friction conditions on the surface quality of truncate cone drawpieces formed from pure titanium (reprinted with permission from [154]; copyright © 2007, Springer-Verlag London Limited).

Hussain et al. [154] investigated the effect of forming parameters on the contact conditions in SPIF of conical drawpieces made from pure titanium. The specimen that was formed by using the MoS_2_ powder and grease showed the best surface quality (no striations on the specimen surface or no metal peeling), and no metal sticking to the tool tip was observed. When the blank was formed without using any lubricant, the blank material adhered to the tool tip and the specimen surface peeled off (Figure 27).

## 8. Numerical Modeling

Supporting the manufacturing processes with finite element-based programs, although quite an expensive process, may contribute to increasing the efficiency of implementing new products in the market. Both large and small companies are most interested in the possibilities for numerical modeling and simulation of manufacturing processes. The virtual models allow one to modernize existing production systems or to implement the manufacturing process without the need to manufacture real tools. Finite element simulations are most efficient for the prediction of elastic-plastic deformations of metallic sheets. Finite element method can be used to predict forming defects, clarify the forming characteristics and improve the forming process. However, due to complicated contact problems involved in the SPIF, efficient approaches for modeling the ISF are still lacking [155].

Many scholars have conducted significant research into the numerical analysis of incremental sheet forming of titanium and Ti-based alloys. Mohanraj and Elangovan [133] performed experimental work and numerical analysis of Ti-6Al-4V incremental sheet forming, considering the spindle speed, tool diameter, feed rate and step size, to study the geometric accuracy and thinning of an aerospace component with complex shape. Simulation results were found to be in agreement with the experimental results, thus showing the applicability of the process in eco-friendly products in low-volume production.

Palumbo et al. [111] conducted FE-based simulations of the SPIF process of non-axisymmetric truncated pyramids with the aim of investigating the effect of both draw angle and tool/step size ratio, taking into account the anisotropic behavior of sheets of Ti-6Al-4V titanium alloy. The analysis of thinning maps and shape errors highlighted that the tool/step size parameter plays a key role in SPIF. The numerical analysis conducted in ABAQUS/Explicit program revealed that large values of the draw angle determined dangerous thinning. Sbayti et al. [156] analyzed hot incremental forming of titanium alloy Ti-6Al-4V denture base by finite element-based simulations. The effects of the forming temperature on the material failure and more particularly on the geometric accuracy of the final product were investigated. It was found that numerical modeling of ISF of denture base by using Ti-6Al-4Vsheets is a feasible solution and it clearly shows the potential for real medical application. Naranjo et al. [157] presented results of the SPIF of Ti-6Al-4V conical drawpieces at different temperatures. Ansys Workbench was used as the numerical program. Based on the recent paper [158], two mesh sizes of hexahedral-type elements were considered for FEM modeling in order to optimize the resolution of the procedure and the computing time. Modeling the Ti-6Al-4V material allowed one to reach a high level of agreement among the forces obtained experimentally and by simulation. It was found that simulation results depend highly on the mesh element size, and a low size must be selected. Moreover, mesh in the corners of squared pyramid makes the simulation process more difficult. Abdeljefi et al. [159] proposed a numerical approach of the SPIF process of titanium T40 sheet by using a simple elastic-plastic material model in Abaqus/implicit© software. The effect of the proposed model on the forming forces and the final geometry was investigated. Saidi et al. [116] investigated the warm incremental process of Ti-6Al-4V sheet based on the use of heat cartridges. The objective was to demonstrate that our low-cost heating system can be used in forming a limit angle similar to that obtained with expensive laser heating. The numerical results were in good agreement with the experimental ones. Piccininni et al. [160] conducted numerical simulations with Tsay–Hill formulation and the strain rate dependent isotropic plasticity law in order to design the SPIF processes of Grade 2 titanium sheets. Finite element models created for simulating both forming processes were revealed to be robust and accurate since a good correlation between numerical and experimental thickness distributions could be obtained. Naranjo et al. [161] tested different plastic behavior hypotheses using a Ti6Al4V alloy to compare the results obtained by FEM with the Ansys program to the experimental tests run to measure the forces applied to the tool. For low strains, a high degree of agreement between the experimentally measured forces and those obtained by simulation was achieved. However, for large strains, most models overpredicted forces obtained experimentally due to the cracks that appeared on the bottom of the cones before the failure occurred.

Most of the studies that refer to FEM for SPIF processes have been traditionally carried out using software with explicit-type solvers like Abaqus [71,162] or LS-Dyna [163,164], or even with software developed especially for this purpose, as in Lagamine [165]. Numerical modeling is used to predict temperature distribution, sheet thickness change, springback, material flow, stress and strain distribution and predictions of forming limit diagrams. FE-based numerical simulation has become an indispensable tool for the prediction of wrinkling and crack occurrence during SPIF. The use of simulation programs requires a thorough knowledge of material behavior and FE programming to develop a suitable numerical analysis for a forming process. It is undeniable that the FE simulations are a huge help in reducing the waste of time and material toward a correct and proper process design. In the future, most numerical research work must be focused on the development of a macroscopic constitutive model based on a physical mechanism, grain-twinning interactions and a crystal plasticity model considering the grain–grain interaction [34].

## 9. Conclusions

Single-point incremental forming plays a key role in transferring state-of-the-art knowledge on manufacturing into innovative solutions of low-volume customized biomedical implants and components in the aerospace industry. This comprehensive review of ISF methods used on titanium and titanium alloys allows the following conclusions to be drawn.

The use of SPIF for biomedical applications is basically limited to the production of implants tailored to individual human needs, mainly dental prosthetics and reconstructive medicine. During the production of custom implants, the SPIF and TPIF methods are limited due to the low dimensional accuracy associated with the residual stresses remaining in the formed material. This research area with regard to titanium and its alloys has not been sufficiently explored.

The geometric accuracy attributed to springback effects and poor surface quality are one of the dominant limits in the implementation of SPIF. Correction of the tool trajectory and heat treatment of components after the forming process allows one to reduce the elastic deformation of the material after cut-out operations and would reduce the pillowing effect.

In SPIF, adhesive wear of the tool predominates. There are insufficient investigations on the synergistic effect of tool rotation direction and rotational speed on the flattening of the surface asperities and quantitative tool wear.

The selection of an appropriate lubricant is a key aspect of the forming process of titanium and its alloys, which exhibit unfavorable tribological properties such as high adhesion and a tendency to adhesive wear that is caused by microscopic transfer of material between the workpiece and tool surfaces. These hot spots are especially important during the deformation of titanium alloys, which are generally only formed under elevated temperature conditions. In such conditions, liquid lubricants cannot be used, and therefore solid lubricants, primarily MoS_2_, are required.

The reduction of forming traces on the surface of implants can be effectively improved by optimizing the tool trajectory and forming strategy, as well as improving the friction conditions. A potential method is to use tools with a textured surface that provide improved lubrication conditions, or tools with a movable spherical tip that freely rolls over the surface of the component being formed.

Research on the phenomenon of friction in conventional SMF processes has shown that biodegradable edible oils can be considered as an alternative to synthetic oils. It seems that this may potentially be a field of research on SPIF carried out in cold-forming conditions that is worth developing.

Due to its crystalline structure, the most commonly used titanium alloy, Ti-6Al-4V, is basically only formed at an elevated temperature. A high temperature, on the one hand, helps to increase the formability of the material but at the same time increases the tendency of the titanium alloy to undergo a severe plastic deformation process (galling). There is insufficient research to determine the balance between SPIF temperature and the quality of the surface finish of the components.

One of the ways to increase the formability of titanium alloys is to treat them at elevated temperatures. Many approaches have been developed for introducing temperature into a workpiece such as electrical heating, laser heating, friction heating or combined electrical and friction heating. The advantage of SPIF with laser heating is lower springback of the formed component. It is easier to control temperature with this method than with friction-assisted SPIF.

The influence of SPIF parameters (i.e., step size, radius of tool tip, feed rate and tool rotational speed) on the surface quality and formability of titanium and its alloys is not identical for materials of different thickness. In many studies based on DoE or Taguchi optimization, sheet thickness is a parameter that is omitted. Future work should focus on exploring such exceptional conditions.

Although titanium and titanium alloys have many desirable characteristics, they have problematic areas as well. These include their affinity to oxygen, hydrogen pickup, susceptibility to certain types of chemical attack, and damage generation arising from the elastic and plastic anisotropy of the hexagonal crystal structure [17]. This makes titanium alloys, especially the most popular Ti-6Al-4V alloy, extremely difficult to deform at ambient temperature. Combinations of working and heat treatment can generate highly preferred crystal orientations, so between the coherent phase boundaries with transformation shears and the anisotropy of the thermal expansion coefficient, substantial residual stresses are common even after a stress relief anneal [3,12,17]. To fully optimize the material property design for particular applications, control of texture and microstructure with SPIF strategies requires predictive understanding of the rules for variant selection during the α→β→α transformations, which are not yet well established [17].

Apart from the knowledge of the microstructure of the material and the related formability, attention should be paid to the strong synergistic effect of forming parameters on the surface finish of the drawpiece and the possibility of obtaining a component with a given degree of deformation. The most critical factors of SPIF process affecting the sheet formability are step size, tool rotational speed, feed rate, tool diameter, sheet thickness and wall angle. Understanding how these parameters influence output quantities (i.e., surface finish, material formability and geometric accuracy) still requires clarity and optimization. First of all, attention should be paid to general observations: (i) the direction of tool rotation in relation to the feed direction is one of the key SPIF parameters influencing the possibility of receiving a titanium drawpiece without the risk of cracking, (ii) samples formed with high values of spindle speed showed poor surface qualities and (iii) the rotation speed is dominant process parameter determining the thermal effect and forming force. A major factor affecting forming forces and quality of the surface finish of drawpieces is the step size. By increasing the spindle speed, a reduction in forming forces was observed [131]. In terms of the surface quality of the formed components, scratches caused by the forming tool can be effectively reduced through the optimization of the toolpath strategy and improvement of lubrication conditions. Proper lubrication is a key factor in enhancing the surface quality at elevated temperatures. When increasing the forming temperature, proper lubrication is a key factor affecting the surface roughness obtained, as well as the oxidation process.

Current findings on the effect of process parameters on output quantities in incremental sheet forming of titanium and Ti-based alloys are limited and sometimes contradictory, which may be due to the various settings of the process parameters and the various geometries of components. Only in terms of the lubrication of the sheet surface, it has been found that MoS_2_ is the most effective lubricant for cold and elevated temperature conditions.

## Figures and Tables

**Table 1:** Typical lubricants used in SPIF of selected materials.

Lubricant	Forming Conditions	Material	Shape of Drawpiece	Reference
OKS 280 lubricant	elevated temperature	Ti-6Al-4V	car door shell	[109]
MOS_2_	elevated temperature	TiAl2Mn1.5	tetragonal pyramid	[92]
MoS_2_	elevated temperature	Ti-6Al-4V	conical frustum/tetragonal pyramid	[83]
graphite	elevated temperature	Ti-6Al-4V	conical frustum/tetragonal pyramid	[83]
boron nitride spray	elevated temperature	Ti-6Al-4V	conical frustum/tetragonal pyramid	[83]
MoS_2_	elevated temperature	Ti-6Al-4V	conical frustum	[77]
MoS_2_, graphite powder, graphite-based and copper-based anti-seize lubricants	elevated temperature	Ti-6Al-4V	conical frustum	[150]
MoS_2_	elevated temperature	Ti-6Al-4V	tetragonal pyramid	[100]
MoS_2_	elevated temperature	Ti-6Al-4V	conical frustum	[86,93,102]
ROCOL copper-based anti-seize compound	elevated temperature	Ti-6Al-4V	conical frustum	[101]
boron nitride	elevated temperature	Ti-6Al-4V	tetragonal pyramid	[104,105]
OKS 280 lubricant	elevated temperature	Ti-6Al-4V	flat components with grooves	[106]
OKS 280 lubricant	elevated temperature	Ti-6Al-4V	truncated cone	[107]
MoS_2_	elevated temperature	Ti-6Al-4V	truncated cone	[151]
carbon-based, dry-film lubricant (Berulit 935)	elevated temperature	Ti-6Al-4V	asymmetric component	[87]
dry graphite	elevated temperature	Ti-6Al-4V	flat component with longitudinal pockets	[89]
MoS_2_	elevated temperature	Grade 2 Ti-6Al-4V	conical frustum	[94,95,96]
Rocol RTD compound	cold-forming conditions	Grade 1	cranial plate	[68]
MoS_2_	cold-forming conditions	pure titanium (grade not specified)	conical frustum	[152]
chlorine-containing forming oil	cold-forming conditions	Grade 1	denture plate	[112]
Nuto 46 hydraulic oil	cold-forming conditions	Grade 2	clavicle implant	[74]
ceramic grease WEICON ASW 040P	cold-forming conditions	Ti Grade 2	facial implant	[71]
MoS_2_	elevated temperature	Ti Grade 2	conical frustum	[153]
MoS_2_	cold-forming conditions	Pure titanium (grade not specified)	conical frustum	[154]

## Data Availability

The data presented in this study are available on request from the corresponding author.
